# Optimal investment and location decisions of a firm in a flood risk area using impulse control theory

**DOI:** 10.1007/s10100-018-0532-0

**Published:** 2018-03-08

**Authors:** Johanna Grames, Dieter Grass, Peter M. Kort, Alexia Prskawetz

**Affiliations:** 1 Vienna University of Technology, Karlsplatz 13/222, 1040 Vienna, Austria; 2 Tilburg University, Tilburg, The Netherlands; 3 University of Antwerp, Antwerp, Belgium; 4 Wittgenstein Centre (IIASA, VID/ÖAW, WU), Institute of Demography, Vienna University of Technology, Vienna, Austria

**Keywords:** Optimal investment, Location choice, Flood, Socio-hydrology, Impulse control theory, Sustainability

## Abstract

Flooding events can affect businesses close to rivers, lakes or coasts. This paper provides an economic partial equilibrium model, which helps to understand the optimal location choice for a firm in flood risk areas and its investment strategies. How often, when and how much are firms willing to invest in flood risk protection measures? We apply Impulse Control Theory and develop a continuation algorithm to solve the model numerically. We find that, the higher the flood risk and the more the firm values the future, i.e. the more sustainable the firm plans, the more the firm will invest in flood defense. Investments in productive capital follow a similar path. Hence, planning in a sustainable way leads to economic growth. Sociohydrological feedbacks are crucial for the location choice of the firm, whereas different economic settings have an impact on investment strategies. If flood defense is already present, e.g. built up by the government, firms move closer to the water and invest less in flood defense, which allows firms to generate higher expected profits. Firms with a large initial productive capital surprisingly try not to keep their market advantage, but rather reduce flood risk by reducing exposed productive capital.

## Introduction

Climate change puts increasing environmental pressure on coastal zones (Turner et al. 1996) and on areas around lakes and rivers Vr̈ösmarty et al. (2000). On top of the list of potential impacts of climate change are effects of sea level rise on coastal cities and effects of extreme events on built infrastructure like floods from heavy precipitation events (Hunt and Watkiss 2011). Floods and other extreme weather events increase economic losses (Easterling et al. 2000). Large-scale flood disasters from recent years have gained attention among decision makers (e.g. businesses). Implementing actions to reduce disaster risks and build flood resilience facing limited resource needs decision support tools (Mechler et al. 2014).

River and Coastal engineers develop risk-analysis techniques for high-level planning and detailed designs using simulation models (Sayers et al. 2002). Economic approaches are cost-effectiveness analyses, multi-criteria analyses, robust-decision-making approaches and dynamic programming (Zwaneveld and Verweij 2014; Eijgenraam et al. 2012, 2014). The most popular tool is cost-benefit analysis applied to cities (Lichfield 1960; Hunt and Watkiss 2011), regions, and countries [e.g. Jonkman et al. (2004)].

There is a number of methods to control floods. Coastal defenses can be e.g. sea walls, beach nourishment, barrier islands or tide gates in conjunction with dykes and culverts. Next to rivers one can construct levees, lakes, dams, reservoirs, bunds, weirs or retention ponds to hold extra water during floods. Moreover, floodways, water gates, diversion channels, temporary barriers or a property level protection can be built. Often flood control measures significantly change the environment and also influence the water system. E.g. levees increase downstream flow and diversion channels redirect water to another area. Both effects increase flood risk nearby. In addition, flood risk increases due to the levee effect (Collenteur et al. 2015), i.e. people and businesses feel save and move closer to the river, and exposed capital accumulates. Other flood control systems like temporary perimeter barriers are not fool proof and can cause unexpected flood damage (Wald 2011). Last, but not least, constructions can restrain the function of a natural flood plain and therefore increase flood risk.

To sum up, installing flood control measures decreases flood risk, but the effect can be significantly reduced when the flood control measure induces feedbacks on the flood hazard or the exposed capital. We include this socio-hydrological feedback mechanismn in our model and study its implications for the system dynamics.

Often investments in flood risk protection measures are done by the government. In this paper we aim to identify the firm’s willingness to pay for flood protection. Furthermore, also actions to reduce flood risk can be taken at the firm-level (Johnson and Priest 2008). Businesses can install their own prewarning systems, choose a more expensive but safer technology for building the production plant, adjust the production process by using a safer construction technology or another type of machines. Last, but not least, more expensive labour agreements attract better human resources.

While our focus is on the firm’s investment decisions we also investigate whether and to which extend the firm level decisions are influenced by investments of the government.

The aim of this paper is to understand investment decisions of firms and their implications on businesses in flood risk areas. Viglione et al. (2014) and Baldassarre and Viglione (2013) developed a conceptual descriptive model to understand the feedbacks of flood risk reduction (i.e. investments in flood defense and moving away from the river) and flood damage from a societal perspective. Grames et al. (2016) introduced an optimal decision framework to investigate the interaction of a society’s investment in flood defense and productive capital. In this paper we consider a partial equilibrium model and try to understand the firm’s investment decisions in its interrelations with the hydrological system. The focus on a firm instead of the whole society allows to specifically analyze a location choice together with the firm’s willingness to pay for flood protection. In contrast to the decisions from a societal point of view, the focus on the firm level also allows us to study the role of firm specific characteristics for the decision process.

A representative firm can have multiple choices: First, it can choose the optimal location for its production plant, second the optimal investment in capital used for production and third, the optimal investment in flood risk reduction measures.

To implement this diverse decision framework our paper rests on three building stones. One building stone consists of so-called capital accumulation models where optimal control theory is applied to determine the firm’s optimal investment behavior over time. This literature starts out with Eisner et al. (1963) and later contributions include Davidson and Harris (1981), Barucci (1998) and Grass et al. (2012).

Another building stone are the impulse control models that consider e.g. dike height optimization, see Chahim et al. (2013). Subject to a water level that increases over time, the decision maker has to decide about the optimal timing and size of the increase of the dike height in order to find an optimal balance between the costs associated with dike height increases and the improved flood protection that results from a dike height increase. This strand of literature abstracts from (firm) investments so that the economic value of the protected land develops exogenously.

The Impulse Control Problem is solved using the impulse maximum principle (Blaquière 1985; Rempala and Zabczyk 1988; Chahim et al. 2012). The general theory of viscosity analysis and quasivariational inequalities [e.g. Barles (1985) and Farouq et al. (2010)] is more consistent, in the sense that it allows more general statements under less restrictive assumptions, covering as many specific cases as possible. However, for the model in this paper the Impulse Maximum Principle seems quite appropriate for an economic interpretation and its numerical calculation.

The underlying paper combines these two approaches, i.e. (impulse) investments have to be undertaken to protect the firm from floods while at the same time the firm establishes an optimal investment pattern that directly influences its economic value.

**Fig. 1:**
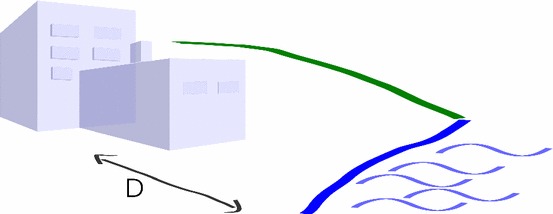
The firm chooses where to build its production plant by choosing the distance to the water

The third building stone is the optimal location choice for the firm’s production plant (Fig. 1) additional to the investment decisions. This location choice is like the choice of technology explained in Brito (2004). A location closer to the water is more profitable in the sense that the water’s infrastructure (transportation, cooling) is easier available and the site is more attractive for the labour force and consumers. But on the other hand being closer to the river implies that the firm faces a larger risk of being flooded.

In location theory Glatte (2015) defines three categories for site selection framework conditions: technical and architectural, economic, and legal, whereas Goette (1994) distinguishes between economic site conditions (sales potential, competitive conditions, infrastructure and transportation costs, labor, monetary conditions), political site conditions (tax legislation, environmental protection, institutional market entry barriers, support of business, political risks), cultural site conditions (differences in language, mentality, religion, and the lack of acceptancy of foreign companies), and geographical site conditions (climate, topography).

Natural hazards are often missing in the site selection literature and the only quantitative methods are cost-benefit analysis or cost-effectivness analysis (Glatte 2015). We introduce a conceptual framework where firms take economic and environmental conditions into account which in turn are affected by the firm’s decisions.

The firm’s decisions are based on an optimization problem, where in a first step the firm is controlling its investments. In a second step, the firm aims to find the optimal location knowing that revenues and costs will depend on the location choice. The firm can only choose its optimal location after having determined the set of optimal investment strategies. The planning horizon is finite, but the firm also considers its salvage value at the end of the planning period. Entrepreneurs do consider only a finite life cycle of a firm. Family businesses may plan in longer terms.

For the firm’s profit maximization only costs are relevant, which can be transferred to monetary values. Consequently, flood damage is measured by so-called direct tangible costs (Merz et al. 2010). We assume the firm to be a production plant with a lot of tangible capital. Direct flood damage reflects the costs of replacing damaged capital (Veen and Logtmeijer 2005).

The aim of the paper is to understand the investment decisions of a representative firm in a flood risk area. We want to identify how much, how often, and when a firm is willing to invest in flood risk protection measures and what the optimal location choice is. Our qualitative model helps to understand feedback mechanisms between the firm’s decisions and the hazard of flooding. In Sect. 2 we explain the general model and its analytical solutions. After discussing the numerical solution of the benchmark model in Sect. 3 we investigate the impact of sustainable planning, the economic situation and the sociohydrological feedbacks in Sect. 4. Section 5 concludes the paper and some detailed derivations are given in the Appendix.

## General model

In this section we set out the general model of investment planning in a flood risk area, setting up the Hamiltonian and Impulse Hamiltonian of the representative firm and deriving necessary conditions for optimally determining flood protection and productive investments. The location choice is done in a second step based on the set of optimal decisions.

### Flood impact

We model a firm located in a flood risk area. The expected flood water level above bankfull

1 $$\begin{aligned} W(t)=W_0 + \eta t, \end{aligned}$$

is some initial water level $W(0)=W_0$ and increases with $\eta$ [cm/year] due to climate change (Eijgenraam Carel 2016). Anthropogenic flood risk protection *H*(*t*), despite decreasing flooding occurrences, may increase flood water levels and consequently flood risk, because e.g. higher dikes make it more difficult for water to stream back in the sea/river after land has been flooded. We model this like Grames et al. (2016) and Viglione et al. (2014) by adding an additional amount of water due to man-made flood risk protection measures $\xi _H H(t)$. $\xi _H$ is the sociohydrological parameter describing the feedback of flood risk protection measures *H*(*t*) to flood risk. The resulting flood intensity $W(t)+\xi _H H(t)$ can be alleviated by increasing the minimum distance to water $D_0$ by the amount *D* for the location of the firm’s production plant in the floodplain with slope $\alpha _D$. Consequently, the flood impact in times of flooding ($W(t)+\xi _H H(t)>H(t)$) is

2 $$\begin{aligned} F_I(W(t),D,H(t))=\frac{W(t)+\xi _H H(t)}{\alpha _D (D_0+D)}. \end{aligned}$$

If the flood impact $F_I(W(t),D,H(t))$ exceeds the current height of flood protection (e.g. dikes, levees) *H*(*t*), damage occurs. According to Chahim et al. (2013) the flood probability $P_F(t)$ [1/year] is given by an initial probability $P_0$ and increases for a higher water level, but decreases with larger flood risk protection measures (i.e. dikes). This leads to the following flooding probability given a scaling parameter $\alpha _F$ [1/cm].

3 $$\begin{aligned} P_F(D,H(t))= & {} P_0 \exp [\alpha _F(F_I(W(t),D,H(t)) - H(t))] \end{aligned}$$

Substituting Eqs. (1) and (2) into Eq. (3) yields Eq. (4), where the socio-hydrological feedback is clearly visible: flooding will reduce the effectiveness of the flood protection by a factor $(1-\frac{\xi _H}{\alpha _D (D_0+D)})$.

4 $$\begin{aligned} P_F(D,H(t))= P_0 \exp \left[ \alpha _F\left( \frac{W(0) + \eta t}{\alpha _D (D_0+D)} - \left( 1-\frac{\xi _H}{\alpha _D (D_0+D)}\right) H(t)\right) \right] \end{aligned}$$

The relative flood damage in case of floods increases with higher flood impact $F_I(t)$ and is expressed as the proportion $F(W(t),D,H(t))\in [0,1]$ of destroyed capital following Grames et al. (2016) and Viglione et al. (2014).

5 $$\begin{aligned} F(W(t),D,H(t))= 1-\exp [-F_I(W(t),D,H(t))] \end{aligned}$$

### Firm’s expected profit

The firm faces a competitive market and produces output *Y*(*t*) choosing the production factors capital *K*(*t*) and the distance *D* to a river or coast in the sense that living closer to the water yields advantages for transport, lowers costs of transporting water to households and industry and is attractive for employees (see Viglione et al. (2014)). The effect of *D* on output is similar to a technological parameter as it scales the firm’s output level for a given set of the other production factors [e.g. Brito (2004)]. We assume a minimum necessary distance to the water body $D_0$. The production function has a Cobb-Douglas form and reads

6 $$\begin{aligned} Y(K(t),D) = \frac{1}{D_0+D}K(t)^{\alpha } \end{aligned}$$

with $\alpha \in [0,1]$. We assume that the firm can sell all its output *Y*(*t*) for a price *p* normalized to 1.

The firm invests $I_K(t)$ in its physical capital which depreciates with rate $\delta _K\in [0,1]$.

7 $$\begin{aligned} {\dot{K}}(t)= I_K(t) - \delta _K K(t) \end{aligned}$$

The costs for capital investment are $\alpha _K I_K(t)^2$ with $\alpha _K$ as a constant scaling parameter.

The value of flood damage is the sum of costs for repairs and cleanup, and costs for lost revenue due to business interruption. First, we assume that repair costs $C_F(K(t), F(t))$ are just as high as the damaged physical capital stock, depending on the impact of flooding $F(t)\in [0,1]$.

8 $$\begin{aligned} C_F(K(t), F(t))=F(t)K(t) \end{aligned}$$

Second, the lost revenue due to business interruption is equal to $P_F(D,H(t)) Y(K(t),D)$. Hence, revenue times probability that no flood occurs reads

9 $$\begin{aligned}{}[1-P_F(D,H(t))]Y(K(t),D). \end{aligned}$$

We assume that everything is repaired immediately after the flooding and production continues with the same capital stock *K*(*t*) and level of flood protection *H*(*t*) after any flooding. Veen and Logtmeijer (2005), Leiter et al. (2009) and Parkatti (2013) are using a similar approach.

To sum up, we can express the expected profit as the difference between expected revenue and expected costs, i.e. investment and damage costs.

10 $$\begin{aligned}&\pi _e(K(t),D,H(t),I_K(t))= (1-P_F(D,H(t)))\big [ Y(K(t),D) - \alpha _K I_K(t)^2\big ] \nonumber \\&- P_F(D,H(t)) C_F(K(t), F(t)) \end{aligned}$$

### Impulse investments in flood defense

Additionally to investments in capital stock *K*, the firm can invest in flood risk protection at the expense of costs $I_H(u_i,H(t))$ to add an amount $u_i>0$ to their flood protection *H*(*t*) at specific points in time $t=\tau _i$. Therefore the firm chooses the optimal number $N\ge 0$ of investments, the optimal timing $\tau _i$ ($i\in \{1,..,N\}$) and the optimal amount $u_i>0$ ($i\in \{1,..,N\}$).

11 $$\begin{aligned} H(\tau _i^+)= H(\tau _i^-)+u_i \end{aligned}$$

holds for $i\in \{1,..,N\}$. Here $H(\tau _i^-)$ is the level of flood risk protection before and $H(\tau _i^+)$ the level of flood risk protection after the *i*th investment.

We model exponential investment costs in flood defense capital following Eijgenraam Carel (2016) with positive constants $\theta _1$, $\theta _2$ and $\theta _3$.

12 $$\begin{aligned} I_H(u,H(\tau ^-))&={\left\{ \begin{array}{ll} (\theta _1+\theta _2u)\exp (\theta _3(H(\tau ^-)+u)) &{} \text {if } u>0\\ 0&{} \text {if } u=0 \end{array}\right. } \end{aligned}$$

For time $t\notin \{\tau _1,...,\tau _N\}$ the flood risk protection capital does not change.

13 $$\begin{aligned} {\dot{H}}(t)= 0 \end{aligned}$$

The firm can invest in flood defense capital during a finite planning period [0, *T*]. The total expected profit considering all types of costs can be displayed as follows using the interest rate *r* to discount future values.

14 $$\begin{aligned} \int _0^T \pi _e(K(\tau ),D,H(\tau ),I_K(\tau )) e^{-r \tau } d\tau - \sum _{i=1}^N I_H(u_i,H(\tau _i)) e^{-r \tau _i} \end{aligned}$$

The value of the firm at the end of the planning horizon *T* is the difference between expected remaining capital $[1-P_F(D,H(T))]K(T)$ and expected damage $P_F(D,H(T))F(D,H(T))K(T)$.

15 $$\begin{aligned} V(K(T),D,H(T))= & {} [1-P_F(D,H(T))]K(T)\nonumber \\&- P_F(D,H(T))F(D,H(T))K(T) \end{aligned}$$

To model not only the expected profit during the planning period we additionally consider the expected value *V*(*K*(*T*), *D*, *H*(*T*)) of the firm after the planning period. Therefore we use the so-called salvage value (Chahim et al. 2013). Note, that the firm does not make any new decisions after the planning period.

16 $$\begin{aligned} \int _T^{\infty } V(K(T),D,H(T)) e^{-r t} dt=\frac{1}{r}e^{-r T}V(K(T),D,H(T)) \end{aligned}$$

### The firm’s optimal decisions

The firm maximizes accumulated discounted profit given an interest rate *r* within a finite planning time horizon expecting floods at unknown times. As a first step it solves the problem for a given value of *D*. It can choose the number *N* of flood defense investments to increase flood risk protection measures by $u_i>0$ and its timings $\tau _i$ during the finite planning period [0, *T*]. It also controls the investment in physical capital $I_K(t)>0$ during the planning period and takes into account the salvage value *V*(*K*(*T*), *D*, *H*(*T*)) weighted with a time preference $\delta _S$.

17a $$\begin{aligned}&\max _{\{u_i,\tau _i,N,I_K(t)\}} \int _0^T \pi _e(K(\tau ),D,H(\tau ),I_K(\tau )) e^{-r \tau } d\tau \nonumber \\&\quad -\sum _{i=1}^N I_H(u_i,H(\tau _i)) e^{-r \tau _i} + \delta _S\frac{1}{r}e^{-r T}V(K(T),D,H(T)) \end{aligned}$$

To summarize, the dynamics of the state variables *K*(*t*) and *H*(*t*) are

17b $$\begin{aligned} {\dot{K}}(t)= & {} I_K(t) - \delta _K K(t) \end{aligned}$$

17c $$\begin{aligned} {\dot{H}}(t)= & {} 0 \quad \text { for } t\notin \{\tau _1,...,\tau _N\} \end{aligned}$$

17d $$\begin{aligned} H(\tau _i^+)= & {} H(\tau _i^-)+u_i \quad \text { for } i\in \{1,...,N\} \end{aligned}$$

and their initial values are $K(0)=K_0$ and $H(0^-)=0$.

As a second step, the firm chooses the optimal location (*D*) for its production plant given the solutions of problem (17a).

We follow the work from Chahim et al. (2012) to derive the necessary optimality conditions for our maximization problem by applying the Impulse Control Maximum Principle (see Appendix B). This way we obtain the optimal paths of the decision variables and the costates. We will describe the intuition and for the detailed results refer to the Appendix A.

The optimal capital investment is as such that the expected revenue stream, including the increase in the salvage value, equals the expected marginal costs. If the production is capital intense, the firm invests intensively in its production capital but slows the investments down with an increasing stock of physical capital, i.e. an extra unit of physical capital is more valuable if the capital stock is (still) small. However, when the expected damage rate is high, the firm will invest less in physical capital. The investment behaviour does not change much if the expected damage rate (possibly amplified by a high water level) is high, but is very sensitive to small changes of low expected damage rates.

A high current and long term value of the physical capital due to a high shadow price and high interest rates motivates the firm to invest in its physical capital, whereas higher investment costs decelerate the accumulation of physical capital. Nonetheless, the firm wants to sustain its capital stock and invests more if the depreciation rate is higher.

The shadow price for physical capital at the end of the planning period equals the difference of the discounted marginal expected output and the expected damage rate.

For investment in flood defense we derive the optimal timing and the amount of investments. For these decisions the shadow price of flood defense capital is crucial. Whenever the benefits of investing in flood defense (i.e. increase in shadow price and expected profit) exceed the costs, the firm will invest in flood risk measures. The amount of investment will be higher if the previous level of flood defense is low and the shadow price of the increased flood defense capital is high. Moreover investment increases if investment costs are low. Still, the cost structure is important: Significantly lower fixed costs could increase the number of investments and therefore decrease the investment amount.

The shadow price for flood defense capital at the end of the planning period is the expected loss from flooding, i.e. the sum of the revenue due to business interruption, the avoided costs at the time of the flood, and the direct damage described by value of repair and cleanup costs. The net present value of the shadow price for flood defense capital increases with expected future loss (i.e. lost profit and damaged capital) augmented with stronger sociohydrological feedbacks and a closer distance to the water. Contrary, if the expected sustained capital at the end of the planning period is high the value decreases.

The number of impulse investments into flood defense capital is rather small (i.e. less than four investments in a feasible planning period) due to fixed costs. Furthermore, the first investment in flood defense is usually early to ensure a low flood hazard for the location of the production plant.

Last, but not least, we find that the optimal level of flood defense can never exceed an upper bound ${\bar{H}}$. Still, this level depends on the propertis of the firm like production capacities and existing capital stock. The upper bound will be lower if the firm locates further away from the river, the sociohydrological feedbacks are small and the initial flooding probability is low.

## Benchmark model

In this section we show the numerical solution of the model and discuss its economic intuition. To derive numerical solutions for our impulse control problem we apply the (multipoint) boundary value approach (Grass 2017). The idea is to solve a boundary value problem (BVP) based on the system dynamics given by the canonical system and update the according boundary conditions at impulse times. A continuation technique is used to continue and find solutions with different number of impulses. The objective values of such solutions are compared and the optimal solution is chosen. Moreover, the continuation alogrithm allows to continue a solution for every model data. Details about the numerical method, which was developed to solve such types of problems, are described in Grass (2017). Details about the application of the numerical method to our proposed model are found in Appendix C. First, we derive the optimal solution for investments depending on the distance *D*. Second, we plot the objective function evaluated at the optimal investment as a function of *D* and locate the maximum with respect to *D*.

We use the following initial conditions. The mean water level above bankfull as well as the flood protection are normalized to zero at the beginning of the planning period. The productive capital initially available for the firm is $$10^8$$ $. The initial flooding probability is 0.001 per year according to Chahim et al. (2013). *D* is referred to a length measure, but scale free. Still, we can exemplify the minimum distance to the water with 5m. All the variables and their initial conditions are listed in Table 1. The parameters are displayed in Table 2. Many parameters (*r*, *A*, $$\alpha $$, $$\alpha _K$$, $$\delta _K$$) are chosen according to standard economic literature, and other parameters ($$\tau _k$$, $$\alpha _P$$) are scaling factors. Most hydrology parameters $$\xi _H$$, $$\alpha _D$$, $$\alpha _F$$ are defined in Viglione et al. (2014). Investment costs in flood protection $$\theta _1$$, $$\theta _2$$, $$\theta _2$$ and natural water level rise $$\eta $$ are introduced in e.g. Chahim et al. (2013) and Eijgenraam Carel (2016). We choose a shorter planning horizon *T* than (Chahim et al. 2013) to reflect a feasible life cycle time of a firm (Lumpkin and Dess 2001). The time discount of the salvage value $$\delta _S$$ is given by $$(1+\delta _L)^T=1+\delta _S$$, where $$\delta _L$$ denotes a standard yearly time preference rate. Note, *r* represents the interest rate of the capital market and is not necessarily equal to the individual time preference rate $$\delta _L$$.

In addition to the the benchmark values we have also listed the values for sensitivity analysis described in the next sections. Note, that our numerical calculations are aimed to provide a qualitative analysis to understand feedbacks and mechanisms within a sociohydrological model of floodings.

**Table 1:** Variables of the model

Variable	Interpretation	Unit		
*t*	Time	[year]		
*K*(*t*)	Productive capital	$$10^6$$ $		
*D*	Firm’s distance to water	$$10^2$$[m]		
$$u_i$$	Height of $$i{\mathrm{th}}$$ increase in flood protection measures	[cm]		
*H*(*t*)	Level of flood protection	[cm]		
$$\tau _i$$	Timing of $$i{\mathrm{th}}$$ investment in flood defense	[year]		
*N*	Number of investments *i*	[ ]		
$$I_H(t)$$	Costs for investment in *H*(*t*)	$$10^6$$ $		
$$\pi _e(t)$$	Firm’s expected profit	$$10^6$$ $		
*Y*(*t*)	Firm’s output	$$10^6$$ $		
$$I_K(t)$$	Investment in physical capital	$$10^6$$ $		
$$C_F(t)$$	Total costs of flooding	$$10^6$$ $		
*W*(*t*)	Water level	[cm]		
*F*(*T*)	Proportion of flooding damage	[0,1]		
$$F_I(T)$$	Flood impact	[]		
$$P_F(t)$$	Flooding probability	[1/year]		
$$\lambda _K(t)$$	Shadow price of physical capital	$$10^6$$ $		
$$\lambda _H(t)$$	Shadow price of flood protection	$$10^6$$ $		

Initial values	Interpretation	Unit	Value	case
$$W_0$$	Initial water level	[cm]	0	
$$K_0$$	Initial productive capital	$$10^6$$ $	100	500
$$H_0$$	Initial flood protection	[cm]	0	200
$$P_0$$	Initial flooding probability	[1/year]	0.001

**Table 2:** Parameters of the model and their units of measurement

Parameter	Interpretation	Unit	Base case	Case study
$$D_0$$	Minimal distance to water	$$10^2$$[m]	0.05	
*T*	End time of planning period	[year]	100	50,150
*r*	Interest rate	[1/year]	0.03	
*A*	Technology	[ ]	1	
$$\alpha $$	Output elasticity of physical capital	[0,1]	0.3	
$$\alpha _K$$	Scale for expected investment in *K*(*t*)	[ ]	0.01	
$$\tau _K$$	Scale for deterministic investment in *K*(*t*)	[ ]	0.01	
$$\delta _K$$	Depreciation rate of *K*(*t*)	[1/year]	0.05	
$$\theta _1$$	Fixed costs for investing in *H*(*t*)	$$10^6$$ $	100	
$$\theta _2$$	Linear costs for investing in *H*(*t*)	$$10^6$$ $/cm	0.5	
$$\theta _3$$	Exponential costs for investing in *H*(*t*)	[$$\ln (10^6$$ $)/cm]	0.005	
$${\tilde{\theta }}_1$$	Transformed fixed costs for investing in *H*(*t*)	$$10^6$$ $	$$\theta _2+\theta _1\theta _3$$	
$${\tilde{\theta }}_2$$	Transformed linear costs for investing in *H*(*t*)	$$10^6$$ $	$$\theta _2\theta _3$$	
$$\eta $$	Increase of water level per year	[cm/year]	0.5	
$$\xi _H$$	Additional rise of the water level due to existing defense capital	[ ]	0.3	0, 0.5
$$\alpha _D$$	Scale of the slope of the floodplain	[ ]	10	
$$\alpha _F$$	Scaling of flooding probability	[1/cm]	0.05	
$$\delta _S$$	Time discount of salvage value	[0,1]	0.1	0, 0.25
$$\alpha _P$$	Approximation parameter for flooding probability	[]	100	

**Fig. 2:**
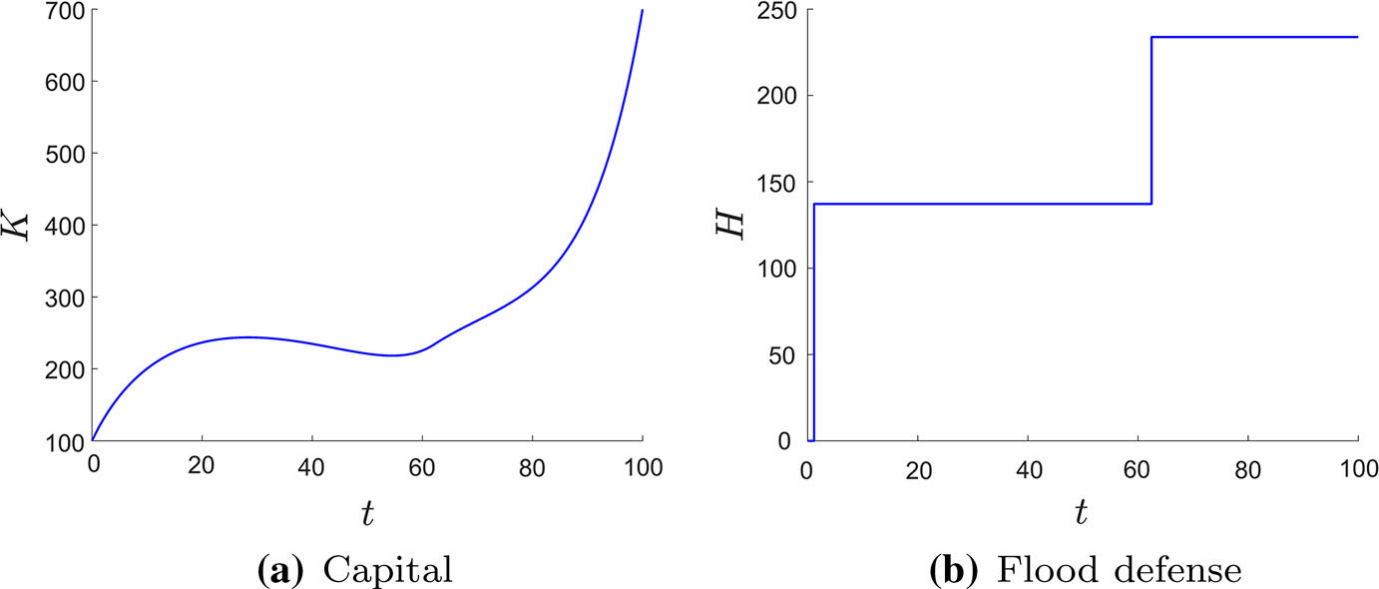
State dynamics in the planning period [0, *T*]. Firm’s capital *K* (**a**) increases only when the flood risk is low as a result of a high flood risk protection standard (**b**)

**Fig. 3:**
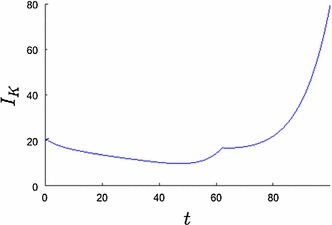
Capital investment $$I_K$$

**Fig. 4:**
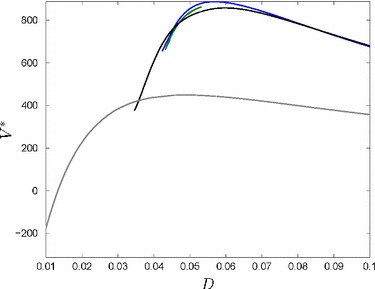
Solution structure given by the objective value $V^*$ depending on *D* for no impulse investments (grey), one impulse investment (black), two impulse investments (blue) and three impulse investments (green). Note, that the objective values do not exist for every value of *D* for each case (color figure online)

The optimal solution is to locate the firm’s production plant rather close to the water (Fig. 4) and to make two impulse investments in flood risk protection measures (Fig. 2b). The dynamics of the capital *K* and the flood defense *H* are displayed in Fig. 2. The first jump occurs very early so that the risk of flooding is very small and the firm can invest in its capital to gain high expected revenues. Since flood risk is increasing with time (Eq. 1) the firm’s investments (Fig. 3) decrease as well. We observe an anticipation effect of the firm, since capital investment increases shortly before the second impulse investment. At the time of an impulse investment $I_H$ the continuous investment $I_K$ jumps too.

The second impulse investment is in the last third of the planning period and just as high as the upper bound ${\bar{H}}$ derived in Eq. (27).

Investments in flood risk protection measures increase economic activity. We can identify that whenever the firm feels saver, it invests more. This is a positive feedback loop and leads to sustainable economic growth, because the firm’s capital is high and flood risk is low.

Moving closer to the water increases production output, but also increases flood risk. Depending on which effect dominates, the expected profit either increases to the left of the peak or decreases to the right of the peak. One can identify this interesting trade-off in Fig. 4, where we find the optimal location of the firm’s production plant (*D*) at the peak of the value function $V^*$ of problem set Eq. (17a).

It is optimal to make two impulse investments (Fig. 4). Investing more often is always slightly worse, because fixed costs occur more often. Investing in flood defense only once or even never would only be better if the firm was located closer to the river, but the objective value would decrease. This would imply that the production output is higher in the beginning and the expected profit much less at the end of the planning horizon because flood risk is increasing dramatically. This also leads to a lower salvage value at the end of the planning horizon.

## Alternative scenarios

In this section we discuss the optimal investment decisions from the perspective of sustainability, the economic setting and the socio-hydrological feedbacks. The firm has three options to adapt to different situations. Firstly, it can choose the number and amount of investment in flood risk protection measures. Secondly, it can choose the investment strategy in its capital within the planning period. Thirdly, it can choose the location for its production plant.

We will compare the different scenarios to the benchmark model to understand which option is most suitable to adapt optimal investment decisions for a different hydrological and economic setting.

### The role of sustainability

Two parameters reflect how important sustainability is for the decision making firm. On the one hand, the salvage value at the end of the planning period is weighted with a certain time preference rate $\delta _S$. On the other hand, the planning horizon *T* is important for the investment decisions. We will discuss both options in detail.

**Fig. 5:**
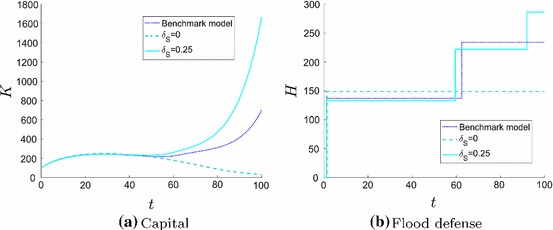
State dynamics for $\delta _S=0$ (dashed blue line), $\delta _S=0.1$ (dotted dark blue line) and $\delta _S=0.25$ (solid light blue line) in the planning period [0, *T*]. If the salvage value of the firm is important (**a**) firm’s capital *K* increases towards the end of the planning horizon and (**b**) firms invest higher amounts and more often in flood risk protection measures

If the value of the firm at the end of the planning period is important ($\delta _S>0$), firms care about flood risk protection measures in the long run and its net present value is significantly higher. The optimal location of the firm’s production plant is at an increased distance to the water body. But the more crucial impact is the investment behavior towards the end of the planning horizon. Fig. 5 shows the time paths of the firm’s capital *K* and the flood defense *H* for different time preference parameters $\delta _S$. The investment behavior in the beginning is rather similar, but for a higher $\delta _S$ the firm invests much more in its productive capital at the end of the planning period. Furthermore, the firm is willing to invest in flood defense more often.

Decision makers in firms with a high time preference rate $\delta _S$ can be e.g. families, entrepreneurs who are confident about a long life time of their product(s) or entrepreneurs who are able to adapt to a changing environment and market demand.

If the firm cannot be sold at the end ($\delta _S=0$) because its product will be outdated and the firm cannot survive on the market anymore, it still invests once to protect itself from floods but tries to make a lot of profits only in the short term. After some time it will neither invest in its own capital ($I_K=0$) nor in flood defense. So the risk of being flooded is much higher.

Even if a firm “only” cares about its own value it is willing to invest in flood risk protection measures and increases economic activity. This is important for the whole region.

**Fig. 6:**
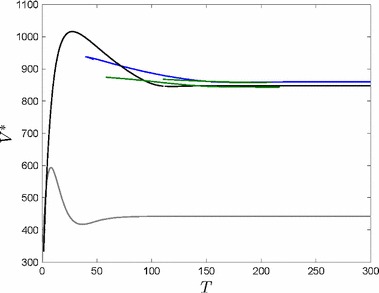
Solution structure given by the objective value $V^*$ depending on the planning horizon *T* for no impulse investments (grey), one impulse investment (black), two impulse investments (blue) and three impulse investments (green) (color figure online)

Firms that do not expect to be on the market for a long time do not care (much) about flood protection. Figure 6 shows the number of impulse investments and the net present value of the firm for various planning horizons *T*.

If the planning period is only a few years firms do not invest in flood defense, and the net present value $V^*$ of the firm is also relatively low.

Firms with a planning horizon around thirty years are most valid. They optimally invest once in flood protection after some years and not in the very beginning.

**Fig. 7:**
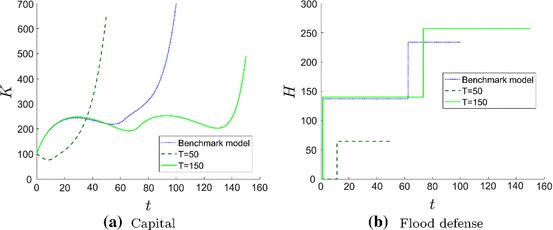
State dynamics for planning horizon $T=50$ (dashed dark green line), $T=100$ (dotted blue line) and $T=150$ (solid light green line). If the planning horizon *T* is longer (**a**) firms invest more in capital *K* even at the beginning and (**b**) firms invest higher amounts and more often in flood risk protection measures

When a firm plans for more than seventy years it optimally invests (at least) twice in flood defense, and the first investment is already very early. Moreover, Fig. 7b shows that the early impulse investment with a planning horizon of 150 years doubles the amount of impulse investment of a firm with a planning horizon of 50 years. Additionally, firms with a longer planning horizon invest more in their capital already at the beginning (see Fig. 7a).

The only disadvantage of investing a lot in flood defense is the necessity to save for these investments and consequently invest less in the firm’s productive capital. This could lead to a regression. Firms would be able to keep investing in their physical capital if e.g. the state government built the flood defense.

To sum up, a sustainable planning process (longer planning horizon) of the firms increases GDP already at the beginning and guarantees a safe environment.

### The economic situation

Depending on the economic situation firms choose different investment strategies. We first analyze a firm in a region where flood protection already exists like e.g. in the Netherlands. Secondly, we investigate the investment decisions of a firm with a high initial capital stock. This can be a company building a production plant in a country with lower prices or a firm with e.g. state subsidy for its company foundation.

**Fig. 8:**
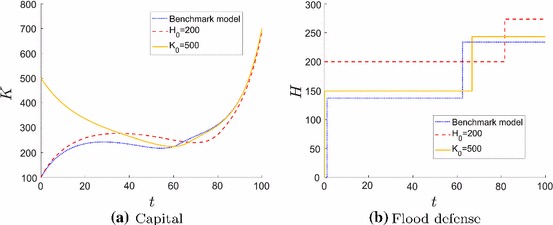
State dynamics for different economic situations: benchmark model with $H_0=0$ and $K_0=10$ (dotted blue line), protected flood plain $H_0=200$ (dashed red line), and capital intense firm $K_0=500$ (solid orange line)

Firms located in a flood risk area where flood protection measures are already installed build their production plant closer to the water and invest in flood defense much later (Fig. 8). Even though the investment behavior in productive capital is similar to the benchmark model, the expected net present value of the firm is higher because the firm plant is safer and closer to the water.

**Fig. 9:**
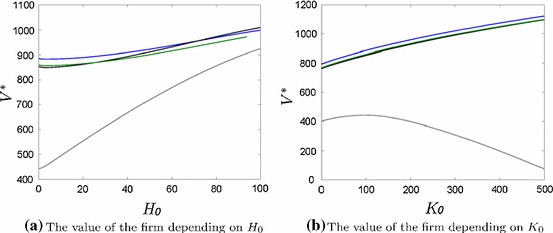
The net present value $V^*$ of the firm increases for higher initial capital stocks. The colors indicate no impulse investments (grey), one impulse investment (black), two impulse investments (blue) and three impulse investments (green) (color figure online)

A firm with high initial productive capital (Fig. 8) does not invest more often in flood protection, but it will invest earlier, i.e. already at $t=0$ and even to a higher extent because the firm has more to loose in case of a flood. Still, its location is only slightly closer to the water. Surprisingly, instead of building extra flood defense, the firm is reducing flood risk by decreasing productive capital $K_0$ to the level in the benchmark scenario. Consequently, the higher value of the firm (Fig. 9b) is only due to higher expected profits at the beginning of the planning period caused by a higher level of initial capital stock $K_0$.

We compare the net present value of a firm depending on initial flood defense $H_0$ and alternatively initial productive capital $K_0$ (Fig. 9). In the first case, investment behavior changes (i.e. for higher $H_0$ less impulse investments are optimal). In the second case, investment behavior does not change (i.e. it is always optimal to invest twice in flood risk protection measures even if the firm could make a large one-time investment at the beginning).

Not investing in flood risk protection measures (grey line in Fig. 9) becomes more attractive for higher $H_0$ because the firm is safer anyways, whereas for a higher productive capital $K_0$ it is less profitable because the exposed capital is larger and therefore possible flood damage is larger. Consequently, flood risk is decreasing for higher (initial) flood defense and increasing for higher (initial) productive capital, since flood risk is defined as the product of flood hazard and exposed capital.

### Sociohydrological feedbacks

Building flood risk protection measures often changes the environment and more specifically the water system. This can cause negative feedbacks for investing in flood defense like e.g. the levee effect or because after a flood it is more difficult for the water to stream back into the river, thereby increasing flood damage. We investigate the effect of these feedbacks on the investment decision of the firm for a scenario with no feedback effects and a scenario with strong feedbacks.

If investment in flood protection affects the water system and increases flood risk, the expected value of the firm decreases dramatically for three reasons: Firstly, firms choose a location much farther away from the water to avoid these negative feedbacks. Secondly, a firm invests less and less often in flood defense, because it increases damage if a flood happens. Thirdly, since the firm is less safe and less profitable because it is located farther away from the water, it will invest less in productive capital, which again leads to a lower production output.

**Fig. 10:**
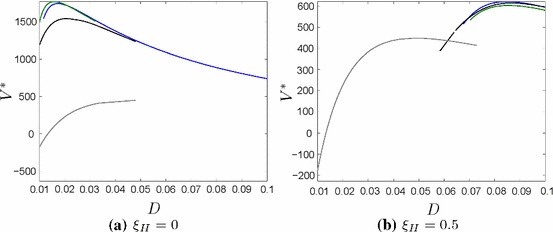
The net present value $V^*$depending on *D* for no impulse investments (grey), one impulse investment (black), two impulse investments (blue) and three impulse investments (green). For high feedbacks ($\xi _H$) it does not pay off to invest in flood defense and will be more profitable to build the production plant further away from the water (color figure online)

Figure 10 shows the value of the firm in case of no feedbacks ($\xi _H=0$) and strong feedbacks ($\xi _H=0.5$). In case of no feedbacks the firm chooses a location much closer to the river and invests three times in flood risk protection measures at almost equal time intervals.

If the hydrological feedbacks are strong the firm builds its premises far away from the water and the value of the firm would not change much if it invests more or less often in flood defense. Still, it is optimal to invest twice. The first investment takes place already after a few years and the second investment is rather at the end of the planning horizon. Interestingly, the total amount of flood defense is almost as high as in the benchmark model, even though the location is much farther away.

We notice that it plays a crucial role if the flood risk protection affects the environment and consequently the water system which is the flood hazard for the firm. We conclude that the damage effect of the flood protection level plays a crucial role in affecting optimal firm behavior.

## Conclusions

This paper provides the investment behaviour and location choice of a firm in a flood risk area within an optimal decision framework. In a first step, the firm chooses timing, number and amount of investments for impulse investments in flood risk protection measures, together with investment in its productive capital within a finite planning period. In a second step, the firm chooses the optimal location for its production plant in the flood risk area.

We present analytical and numerical solutions and analyse variations of these solutions under different parameterizations of the model. Sustainable investment planning of the firm doees not lead only to a safer environment with less flood risk, but also to economic growth both in the short and the long run. If the area is already protected against floods, firms still invest in flood defense, but less. And if the firm is more capital intensive potential damage is larger, but the timing and amount of impulse investments do not change.

Anthropogenic flood risk reduction can affect the environment resulting in changes of the water system and consequently again increase flood risk due to negative feedbacks. In this case, production output is much less and the firm decides to build its production far away from the water.

So far we have presented a qualitative numerical analysis of our model set up. It would be interesting in further work to numerically calibrate our model with empirical data from case studies.

Other topics of future research could be to introduce depreciation and maintenance of flood risk protection measures or to simulate random flooding events (e.g. Poisson distribution) like Grames et al. (2016) or Viglione et al. (2014) and imply them like shocks in the model of Kuhn et al. (2017).

Furthermore, our partial equilibrium setup reflecting the firm’s decisions could be extended to a general equilibrium framework that also models both the household’s behavior and government policies endogenously, in addition to the firm’s optimal decisions. This allows for an analysis of the society as a whole given all the economic interactions.

Last but not least one could apply the method of impulse control to the decision framework of a social planner who represents the whole society and can include e.g. environmental quality in their objective function.

## A Details of the firm’s optimal decisions

In addition to Sect. 2.4 we provide more detailed insights about the optimal decisions of the firm. To still enable smooth reading we present the derivations of the optimal decisions in Appendix B.

### A.1 Optimal capital investment

The optimal dynamics of the investment in physical capital between the impulse investments is given by Eq. (18). The firm increases investments in physical capital if the interest rate is high and the depreciation rate is high. If the expected output per physical capital is already high, the firm slows down investment, whereas investment is increased for a higher capital stock or a higher shadow price of the capital stock. Moreover, the elasticity of physical capital in the production function has a negative impact on the investment decision. Marginal investment increases if the expected damage rate increases. Investment decreases if the water level rises.

18 $$\begin{aligned} {\dot{I}}_K(t)= & {} I_K(t)\left[ r+\delta _K-\frac{\alpha }{\lambda _K} \frac{(1-P_F(D,H(t)))Y(K(t),D)}{K(t)}\right. \nonumber \\&\left. +\frac{1}{\lambda _K}P_F(D,H(t))F(D,H(t))+\frac{\alpha _F}{\lambda _K}\frac{P_F(D,H(t))}{1-P_F(D,H(t))}\frac{\eta }{\alpha _D D}\right] \end{aligned}$$

Solving the differential equation from the first order conditions and the transversality condition yields the net present value for the expected optimal investment in physical capital $I_K(t)$ at time *t*.

19 $$\begin{aligned}&(1-P_F(D,H(t)))I_K(t) = \nonumber \\&\frac{1}{2\alpha _K }\int _t^T \frac{\alpha [1-P_F(D,H(s))]Y(K(s),D)-P_F(D,H(s))F(D,H(s))K(s)}{K(s)} e^{-(r+\delta _k)(s-t)}ds \nonumber \\&+ e^{-(r+\delta _K)(T-t)}\frac{1}{r} \frac{\alpha }{2\alpha _K}[1-P_F(D,H(T))]\frac{Y(K(T),D)}{K(T)}-P_F(D,H(T))F(D,H(T))\nonumber \\ \end{aligned}$$

Given lower investment costs ($2\alpha _K$) the firm invests more in physical capital. Additionally, more expected output per capital in the future in a more capital intense production ($\alpha$) increases the investment. On the other hand, a higher expected damage rate decreases the optimal investment. To conclude, expression (19) shows that the productive investment rate is determined as such that the resulting expected revenue stream, including the increase in the salvage value, due to a marginal investment, equals the expected marginal investment costs.

At the end of the planning period *T* the optimal investment rate will be equal to the difference between expected outcome per capital and expected damage per capital.

20 $$\begin{aligned}&(1-P_F(D,H(T)))I_K(T) \nonumber \\&\quad =\frac{1}{r} \frac{\alpha }{2\alpha _K} [1-P_F(D,H(T))]\frac{Y(K(T),D)}{K(T)} -P_F(D,H(T))F(D,H(T))\nonumber \\ \end{aligned}$$

### A.2 Shadow prices

Analogous to the derivation of Eq. (19) we obtain the net present value of the shadow price for investment in flood defense.

21 $$\begin{aligned} \lambda _H= & {} \int _t^T\big (P_F(D,H(s))\alpha _F \left( 1-\frac{\xi _H}{\alpha _D(D_0+D)}\right) [Y(K(s),D)\nonumber \\&-\,\alpha _K I_K(s)^2-F(D,H(s))]\nonumber \\&-\,P_F(D,H(s))(1-F(D,H(s)))\left( \frac{\xi _H}{\alpha _D(D_0+D)})\right) e^{r(t-s)}ds \nonumber \\&-\,e^{r(t-T)} \frac{1}{r} P_F(D,H(T))\alpha _F \left( 1-\frac{\xi _H}{\alpha _D(D_0+D)}\right) [Y(K(T),D)\nonumber \\&-\,\alpha _K I_K(T)^2-F(D,H(T))] \nonumber \\&-\,P_F(D,H(T))(1-F(D,H(T)))\left( \frac{\xi _H}{\alpha _D(D_0+D)}\right) \end{aligned}$$

The shadow price of flood protection increases with expected future losses (i.e. lost profit and damaged capital) and decreases with expected sustained capital. The shadow price $\lambda _H$ increases if sociohydrological feedbacks are more intense and if the firm decided to build the production plant closer to the water.

The transversality conditions Eq. (35) yield expressions for the shadow prices at time $T^+$.

22a $$\begin{aligned} \lambda _K(T^+)= & {} \frac{1}{r} \alpha \frac{[1-P_F(H(T^+),D)]Y(K(T^+),D)}{K}-P_F(H(T^+),D)F(H(T^+))\nonumber \\ \end{aligned}$$

22b $$\begin{aligned} \lambda _H(T^+)= & {} \frac{1}{r} P_F(H(T^+),D) \Bigg [\alpha _F\left( 1-\frac{\xi _H}{\alpha _D (D_0+D)}\right) (Y(K(T^+),D)-\alpha _K I_K(T^+)^2\nonumber \\&+ F(H(T^+),D)K(T^+)) \Bigg ] \end{aligned}$$

The shadow price for physical capital at the end of the planning period (22a) equals the discounted difference of expected output per capital and the expected damage rate. The shadow price for flood defense capital at $T^+$ is the expected loss from flooding, i.e. the sum of the revenue due to business interruption $P_F(H,D) Y(K,D)$, the avoided costs at the time of the flooding $-P_F(H,D)(\alpha _K I_K^2)$ and the direct damage as the value of repair and cleanup costs $P_F(H,D)F(H)K$.

### Optimal flood defense

Firms invest in flood defense when marginal costs equal marginal gain at the jump point $\tau _i$ ($u_i>0$). This is shown by the first order impulse conditions

23 $$\begin{aligned} \lambda _H(\tau _i^+)=\frac{\partial I_H}{\partial u}(u_i,H(\tau _i^-))=({\tilde{\theta }}_1+{\tilde{\theta }}_2 u_i)\exp (\theta _3(H(\tau ^-)+u_i)), \end{aligned}$$

where ${\tilde{\theta }}_1:=\theta _2+\theta _1 \theta _3$ and ${\tilde{\theta }}_2:=\theta _2 \theta _3$, and the jumping condition,

24 $$\begin{aligned} \lambda _H(\tau _i^+)-\lambda _H(\tau _i^-)= & {} \frac{\partial I_H}{\partial H}(H(\tau _i^-),u_i,\lambda _H(\tau _i^+),\tau _i) \nonumber \\= & {} ({\tilde{\theta }}_3+{\tilde{\theta }}_2 u_i)\exp (\theta _3(H(\tau ^-)+u_i)), \end{aligned}$$

where $${\tilde{\theta }}_3:=\theta _1 \theta _3$$.

At the jump points, i.e. when the firm invests in flood protection, the increase in expected profit should equal the investment costs or be higher at the initial point in time provided the firm invests in flood protection at this time.

25 $$\begin{aligned} \pi _e(K(\tau _i),D,H(\tau _i^+),I_K(\tau _i^+)) -\pi _e(K(\tau _i),D,H(\tau _i^-),I_K(\tau _i^-)) \nonumber \\ + \lambda _K(\tau _i^+) [I_K(\tau _i^+)-\delta _K K(\tau _i^+)]-\lambda _K(\tau _i^-) [I_K(\tau _i^-)-\delta _K K(\tau _i^-)]\nonumber \\ -rI_H(u_i,H(\tau _i^-)) = {\left\{ \begin{array}{ll} >0 &{} \text {if } \tau _i=0\\ =0 &{} \text {if } \tau _i\in (0,T)\\ <0 &{} \text {if } \tau _i=T \end{array}\right. } \end{aligned}$$

If we assume that for every planning horizon *T* there exists a unique optimal solution for our problem (17a) with a finite number of jumps, we can derive the optimal impulse control value $u_i$ (Grass and Chahim 2012).

With the necessary condition Eq. (23) we obtain an implicit function of the optimal value $u_i$ at time $\tau _i$.

26 $$\begin{aligned} H(\tau _i^-)+u_i=-\ln (({\tilde{\theta }}_1+{\tilde{\theta }}_2 u_i)^{\frac{1}{\theta _3}})+\ln (\lambda _H(\tau _i^+)^{\frac{1}{\theta _3}}) \end{aligned}$$

$\frac{\partial ^2 IHam}{\partial u^2}(u_i,H(\tau _i^-))$ is always negative for $u\ge 0$ and ensures that $u_i$ is optimal.

Furthermore, we are able to identify an upper bound ${\bar{H}}$ for the level of flood defense capital *H* given an optimal solution. The detailed derivation is found in Appendix 5.1.

27 $$\begin{aligned} {\bar{H}}=\frac{\ln \big (\frac{P_0\alpha _F(Y+K)}{r\theta _1\theta _3}\big )+\alpha _F\frac{W_0+\eta T^+}{\alpha _D (D_0+D)}}{\theta _3+\alpha _F\left( 1-\frac{\xi _H}{\alpha _D (D_0+D)}\right) } \end{aligned}$$

So we know ${\bar{H}}>H(T^+)$. Since the water level (1) is increasing it holds that $H(T^+)>H(t)$ for all $t\in [0,T]$. ${\bar{H}}$ still depends on *Y*(*K*, *D*) and *K*, and can therefore vary for different properties of the firm.

${\bar{H}}$ increases for a higher flood risk. A higher flood risk can be caused by a higher flood hazard ($P_F(t)$), i.e. the initial flooding probability $P_0$ or the water level increases or the firm is located closer to the water in a flatter flood plain. Defense capital will also be higher if the damage resulting from a flood is higher, which is the case if exposed capital (*K*(*t*)) is higher. Flood risk also increases if exposed capital increases. The only parameter to lower ${\bar{H}}$ are the costs of investments $I_H$ in flood defense.

## B Additional derivations and explanations

### B.1 Necessary Optimality Conditions

We follow the work from Chahim et al. (2012) to derive the necessary optimality conditions for our maximization problem. We use the current value Hamiltonian form to incorporate the discounting.

To apply the Impulse Control Maximum Principle the functions $\pi _e(t)$ and $I_H(u_i,H(t))$ should be continuously differentiable in *H* and $u_i$ on ${\mathbb {R}}_+$, and $\frac{1}{r} \pi _e(T)$ should be continuously differentiable in *K*(*T*) and *H*(*T*) on ${\mathbb {R}}_+$. Furthermore, $I_H(u_i,H(\tau ^-))$ should be continuous in $\tau$.

The maximization problem displayed in Eq. (17a) yields the following current value Hamiltonian

28 $$\begin{aligned} Ham(K,I_K,\lambda _K,t)=\pi _e(K(t),D,H(t),I_K(t)) + \lambda _K [I_K(t) - \delta _K K(t)] \end{aligned}$$

and the following current value Impulse Hamiltonian.

29 $$\begin{aligned} IHam(H,u,\lambda _H,t)= -I_H(u,H(t)) + \lambda _H u \end{aligned}$$

The necessary optimality conditions in our model are as follows. For all $t\notin \{\tau _1,...,\tau _N\}$ it holds that

30 $$\begin{aligned} \frac{\partial Ham}{\partial I_K}(K,I_K,\lambda _K,t)= & {} 0 \end{aligned}$$

31a $$\begin{aligned} \frac{\partial Ham}{\partial K}(K,I_K,\lambda _K,t)= & {} r\lambda _K-{\dot{\lambda }}_K \end{aligned}$$

31b $$\begin{aligned} \frac{\partial Ham}{\partial H}(K,I_K,\lambda _K,t)= & {} r\lambda _H-{\dot{\lambda }}_H \end{aligned}$$

and for any $u\ge 0$

32 $$\begin{aligned} \frac{\partial IHam}{\partial u}(H,0,\lambda _H,t)u \le 0. \end{aligned}$$

For the impulses $t\in \{\tau _1,...,\tau _N\}$ and non-negative heightenings $u\ge 0$ the following holds.

33 $$\begin{aligned} \frac{\partial IHam}{\partial u}(H(\tau _i^-),u_i,\lambda _H(\tau _i^+),\tau _i)= 0 \end{aligned}$$

34a $$\begin{aligned} \lambda _H(\tau _i^+)-\lambda _H(\tau _i^-)=-\frac{\partial IHam}{\partial H}(H(\tau _i^-),u_i,\lambda _H(\tau _i^+),\tau _i) \end{aligned}$$

34b $$\begin{aligned} \lambda _K(\tau _i^+)-\lambda _K(\tau _i^-)=-\frac{\partial IHam}{\partial K}(H(\tau _i^-),u_i,\lambda _H(\tau _i^+),\tau _i)=0 \end{aligned}$$

At the end of the time interval the transversality conditions

35a $$\begin{aligned} \lambda _K(T^+)= & {} \frac{1}{r}\frac{\partial \pi _e}{\partial K}(K(T^+),H(T^+)) \end{aligned}$$

35b $$\begin{aligned} \lambda _H(T^+)= & {} \frac{1}{r}\frac{\partial \pi _e}{\partial H}(K(T^+),H(T^+)) \end{aligned}$$

hold with $K(T^+)=K(T)$ and $H(T^+)=H(T)$ if there is no jump at time *T* and $\tau _1<\tau _2<\cdots <\tau _N\le T$.

### B.2 Derivation of equations for Sect. 2.4

From the condition (30) we obtain

36 $$\begin{aligned} \lambda _K=(1-P_F(D,H))2\alpha _KI_K. \end{aligned}$$

Taking the logarithm and time derivative and combining them with the result from (31a) leads to the following optimal dynamics of the investment in physical capital between the impulse investments shown in (18).

Solving the differential equation from condition (31a) for $\lambda _K$, using the transversality condition (22a) and Eq. (36) yields the net present value for the expected optimal investment in physical capital $I_K(t)$ expressed in Eq. (19).

The necessary condition (31b) yields the dynamics of the shadow price for investment in flood defense capital.

37 $$\begin{aligned} {\dot{\lambda }}_H= & {} r\lambda _H-P_F(D,H)\alpha _F \left( 1-\frac{\xi _H}{\alpha _D(D_0+D)}\right) [Y(K,D)-\alpha _K I_K^2-F(D,H)]\nonumber \\&+P_F(D,H)(1-F(D,H))\left( \frac{\xi _H}{\alpha _D(D_0+D)}\right) \end{aligned}$$

We can solve that differential equation (37) using the transversality condition (22b) to obtain Eq. (21).

### B.3 Derivation of ${\bar{H}}$

We know investment is only optimal if marginal gain (22b) is at least equal to marginal costs (23) at time $T^+$ . The resulting equation

38 $$\begin{aligned}&\frac{1}{r} P_F(D,H) \bigg [\alpha _F\left( 1-\frac{\xi _H}{\alpha _D (D_0+D)}\right) \big [Y-\alpha _K I_K^2+ F(D,H)K\big ]\nonumber \\&\quad -\frac{\xi _H}{\alpha _D (D_0+D)}[1-F(D,H)]K \bigg ] \nonumber \\&\quad = ({\tilde{\theta }}_1+{\tilde{\theta }}_2 u_i)\exp (\theta _3(H(\tau ^-)+u_i)) \end{aligned}$$

ensures that an upper bound ${\bar{H}}$ exists, because the left hand / the right hand side of the equation converges to 0 / $\infty$ for $H\rightarrow \infty$, respectively. We define $A:=\alpha _F(1-\xi _H)Y+ \alpha _F(1- \xi _H) F(D,H)K -\frac{\xi _H}{\alpha _D (D_0+D)}[1-F(D,H)]K$ and find ${\bar{A}}\ge A$ at $T^+$ with ${\bar{A}}=\alpha _F(1-\xi _H)(K+Y)$. ${\bar{A}}$ is constant at $T^+$. Since we know that ${\bar{H}}$ still holds for increased marginal gain or decreased marginal costs, we can reduce Eq. (38) to

39 $$\begin{aligned}&\frac{1}{r} P_0 \exp \Big ({\alpha _F\Big (\frac{W_0+\eta T^+}{\alpha _D (D_0+D)}-\Big (1-\frac{\xi _H}{\alpha _D (D_0+D)}\Big ){\bar{H}}}\Big ) \alpha _F\Big (1-\frac{\xi _H}{\alpha _D (D_0+D)}\Big )(Y+K) \nonumber \\&\quad =\theta _1\theta _3\exp (\theta _3{\bar{H}}) \end{aligned}$$

and derive $${\bar{H}}$$.

## C Numerical solution

To apply the continuation algorithm introduced in Sect. 3 we have to derive the model dynamics explicitely. For convenience we do not write the time argument *t* to the dynamic variables *K*, *H*, $\lambda _K$, $\lambda _H$, $I_K$.

To avoid a positive product caused by two negative factors $(1-P_F)$ and $(Y-\alpha _K I_K^2)$ and to ensure that $(1-P_F)\in [0,1]$ we approximate the term $(1-P_F)$ with $\frac{1}{1+\alpha _P P_F}$.

We use the following short notations.

40a $$\begin{aligned} Y(K,L,D)= & {} K^{\alpha }\frac{1}{(D_0+D)} \end{aligned}$$

40b $$\begin{aligned} \pi _e(K,D,H,I_K)= & {} \frac{1}{1+\alpha _P P_F} \big [Y(K,D) - \alpha _K I_K^2 \big ] \nonumber \\&- P_F(H,D) F(H,D) K \end{aligned}$$

40c $$\begin{aligned} P_F(H,D)= & {} P_0\exp \left[ \alpha _F \left( \frac{W_0+\eta t}{\alpha _D (D_0+D)} - \left( 1-\frac{\xi _H}{\alpha _D (D_0+D)}\right) H\right) \right] \nonumber \\ \end{aligned}$$

40d $$\begin{aligned} F(H,D)= & {} 1-\exp {\left( -\frac{W+\xi _H H}{\alpha _D (D_0+D)}\right) } \end{aligned}$$

40e $$\begin{aligned} I_H(u,H(\tau ^-))= & {} (\theta _1+\theta _2u)\exp (\theta _3(H(\tau ^-)+u)) \end{aligned}$$

Note, Eq. (40e) is only used for *u* strictly positive.

We can summarize the canonical system dynamics for $t\in (\tau _{i-1},\tau _i)$ with $i\in \{1,...,N+1\}$.

41a $$\begin{aligned} {\dot{K}}= & {} I_K - \delta _K K \end{aligned}$$

41b $$\begin{aligned} {\dot{H}}= & {} 0 \end{aligned}$$

41c $$\begin{aligned} {\dot{\lambda }}_K= & {} (r+\delta _K)\lambda _K-\alpha \frac{\frac{1}{1+\alpha _P P_F}Y(K,L,D)}{K}+P_F(H,D)F(H,D) \end{aligned}$$

41d $$\begin{aligned} {\dot{\lambda }}_H= & {} r\lambda _H-P_F(D,H)\alpha _F \left( 1-\frac{\xi _H}{\alpha _D(D_0+D)}\right) [Y(K,D)-\alpha _K I_K^2-F(D,H)]\nonumber \\&+P_F(D,H)(1-F(D,H))\left( \frac{\xi _H}{\alpha _D(D_0+D)}\right) \end{aligned}$$

41e $$\begin{aligned} {\dot{I}}_K= & {} I_K\left[ r+\delta _K-\frac{\alpha }{\lambda _K} \frac{\frac{1}{1+\alpha _P P_F})Y(K,L,D)}{K}+\frac{1}{\lambda _K}P_F(H,D)F(H,D)\right. \nonumber \\&\left. +\frac{\alpha _F}{\lambda _K}\frac{P_F(H,D)}{1-P_F(H,D)}\frac{\eta }{\alpha _D (D_0+D)}\right] \end{aligned}$$

Moreover, we rewrite the conditions for the jump points $\tau _i$ with $i\in \{1,...,N\}$.

42a $$\begin{aligned}&H(\tau _i^+)- H(\tau _i^-)-u_i=0 \end{aligned}$$

42b $$\begin{aligned}&\lambda _H(\tau _i^+)-({\tilde{\theta }}_1+{\tilde{\theta }}_2 u_i)\exp (\theta _3(H(\tau ^-)+u_i))=0 \end{aligned}$$

42c $$\begin{aligned}&\lambda _H(\tau _i^+)-\lambda _H(\tau _i^-)-({\tilde{\theta }}_3+{\tilde{\theta }}_2 u_i)\exp (\theta _3(H(\tau ^-)+u_i))=0 \end{aligned}$$

42d $$\begin{aligned}&\pi _e(K(\tau _i),H(\tau _i^+),I_K(\tau _i^+)) -\pi _e(K(\tau _i),H(\tau _i^+),I_K(\tau _i^-)) \nonumber \\&\quad + \lambda _K(\tau _i^+) [I_K(\tau _i^+)-\delta _K K(\tau _i^+)]-\lambda _K(\tau _i^-) [I_K(\tau _i^-)-\delta _K K(\tau _i^-)]\nonumber \\&\quad -rI_H(u_i,H(\tau _i^-)) =0 \end{aligned}$$

We solve the conditions for every interval assuming $0<\tau _1<\tau _2<cdots<\tau _N<\tau _{N+1}=T$. The starting values are

43a $$\begin{aligned} K(0)= & {} K_0 \end{aligned}$$

43b $$\begin{aligned} H(0^-)= & {} 0 \end{aligned}$$

and at the end *T* the transversality conditions have to hold. Note, that here the time argument for all the dynamic variables is time *T*.

43c $$\begin{aligned} \frac{1}{r} \left[ \alpha \frac{[1-P_F(H,D)]Y(K,D)}{K}-P_F(H,D)F(H)\right] -\lambda _K =0 \nonumber \\ \end{aligned}$$

43d $$\begin{aligned} \frac{1}{r} P_F(H,D) \left[ \alpha _F\left( 1-\frac{\xi _H}{\alpha _D (D_0+D)}\right) (Y(K,D)-\alpha _K I_K^2 + F(H,D)K) \right] -\lambda _H = 0 \nonumber \\ \end{aligned}$$
